# Supplementary material for: Non-linear relationships between auditory mismatch responses and the inharmonicity of complex sounds
Source: Sci Rep. 2026 Mar 3;16:11836. doi: 10.1038/s41598-026-41129-7 (PMC13066439; doi:10.1038/s41598-026-41129-7)
Supplement: Supplementary file 1 — Supplementary Material 1 [file 41598_2026_41129_MOESM1_ESM.pdf]

Supplementary materials

Supplementary Table 1. One-sample, two-sided t-tests of mean MMN and P3 amplitudes for all studied jitter conditions, tested against 0.

Condition	*M[95%CI]*	*t*	*df*	*p*	p_{adj}
MMN amplitude					
j0	-1.17[-1.61, -0.74]	-5.45	34	<.001	<.001
j1	-1.49[-1.86, -1.11]	-8.08	34	<.001	<.001
j2	-1.13[-1.51, -0.76]	-6.18	34	<.001	<.001
j3	-1.22[-1.66, -0.78]	-5.61	34	<.001	<.001
j4	-0.91[-1.28, -0.54]	-4.97	34	<.001	<.001
j5	-0.37[-0.62, -0.12]	-2.97	34	0.005	0.007
j6	-0.39[-0.73, -0.06]	-2.39	34	0.023	0.026
j7	-0.32[-0.64, -0.01]	-2.08	34	0.045	0.045
P3 amplitude					
j0	0.93[0.54, 1.32]	4.83	34	<.001	<.001
j1	0.51[0.12, 0.89]	2.69	34	0.011	0.011
j2	1.01[0.6, 1.42]	5.00	34	<.001	<.001
j3	0.91[0.55, 1.27]	5.14	34	<.001	<.001
j4	0.99[0.57, 1.41]	4.75	34	<.001	<.001
j5	0.79[0.51, 1.07]	5.71	34	<.001	<.001
j6	0.54[0.19, 0.88]	3.17	34	0.003	0.004
j7	0.58[0.24, 0.92]	3.48	34	0.001	0.002

Note: *M[95%CI]* indicates mean and its 95% confidence interval; *t* indicates t-test statistic, *df* indicates degrees of freedom; *p* indicates uncorrected p-value; p_{adj} was calculated using Benjamini & Hochberg correction for multiple comparisons; results were considered significant if p_{adj} < 0.05; j0 is the control, harmonic condition and j1-7 indicate subsequent inharmonic conditions.

Supplementary Table 2. ANOVA results for the effect of inharmonicity on MMN and P3 amplitudes and latencies.

Effect	DFn	DFd	SSn	SSd	F	p	ges	W	p_{GG}
MMN amplitude									
(Intercept)	1	34	214.39	121.52	59.99	<.001	0.41		
jitter	7	238	50.60	188.55	9.12	<.001	0.14	0.17*	<.001
P3 amplitude									
(Intercept)	1	34	170.46	106.25	54.54	<.001	0.35		
jitter	7	238	10.81	206.97	1.78	0.093	0.0	0.37	
**MMN latency**									
(Intercept)	1	34	6.85	0.05	4363.87	<.001	0.94		
jitter	7	238	0.01	0.35	0.82	0.57	0.02	0.2*	0.538
**P3 latency**									
(Intercept)	1	34	18.40	0.09	7223.28	<.001	0.98		
jitter	7	238	0.01	0.36	1.22	0.29	0.03	0.23*	0.299

Note: DFn - degrees of freedom of the numerator; DFd - degrees of freedom of the denominator; SSn - sum of squares of the numerator; SSd - sum of squares of the denominator; F - F statistic; p - uncorrected p-value; ges - generalized eta squared; W - Mauchly's W statistic; p_{GG} - Greenhouse-Geiser corrected p-value.

Supplementary Table 3. Pairwise contrasts of mismatch negativity amplitude across jitter conditions.

Jitter pair	Estimate	SE	df	t	p
j0 - j1	0.3108	0.213	238	1.461	0.827
j0 - j2	-0.0416	0.213	238	-0.196	1.000
j0 - j3	0.0435	0.213	238	0.204	1.000
j0 - j4	-0.2676	0.213	238	-1.258	0.913
j0 - j5	-0.8093	0.213	238	-3.804	0.004
j0 - j6	-0.7823	0.213	238	-3.677	0.007
j0 - j7	-0.8521	0.213	238	-4.005	0.002
j1 - j2	-0.3524	0.213	238	-1.656	0.715
j1 - j3	-0.2673	0.213	238	-1.256	0.914
j1 - j4	-0.5784	0.213	238	-2.718	0.122
j1 - j5	-1.1201	0.213	238	-5.264	<.001
j1 - j6	-1.0931	0.213	238	-5.137	<.001
j1 - j7	-1.1629	0.213	238	-5.465	<.001
j2 - j3	0.0851	0.213	238	0.400	1.000
j2 - j4	-0.2260	0.213	238	-1.062	0.964
j2 - j5	-0.7677	0.213	238	-3.608	0.009
j2 - j6	-0.7407	0.213	238	-3.481	0.014
j2 - j7	-0.8105	0.213	238	-3.809	0.004
j3 - j4	-0.3111	0.213	238	-1.462	0.827
j3 - j5	-0.8528	0.213	238	-4.008	0.002
j3 - j6	-0.8258	0.213	238	-3.881	0.003
j3 - j7	-0.8956	0.213	238	-4.209	0.001
j4 - j5	-0.5417	0.213	238	-2.546	0.182
j4 - j6	-0.5147	0.213	238	-2.419	0.237
j4 - j7	-0.5845	0.213	238	-2.747	0.114
j5 - j6	0.0270	0.213	238	0.127	1.000
j5 - j7	-0.0428	0.213	238	-0.201	1.000
j6 - j7	-0.0698	0.213	238	-0.328	1.000

Note: Pairwise comparisons were conducted using estimated marginal means (EMMs) to evaluate differences in mismatch negativity (MMN) amplitude between jitter conditions (j0–j7). The table reports: Estimate - the contrast estimates (in μV); SE - standard error of the estimate; df - degrees of freedom; t - t statistic; p - p-values adjusted for multiple comparisons using the Tukey method.

Supplementary Table 4. Model comparison of MMN and P3 amplitude as a function of jitter.

Model degree	df	AIC	BIC	logLik	Test	L.Ratio	p
MMN amplitude - linear and polynomial models							
0 (Intercept only)	3	736.13	746.63	-365.07		-	-
1 (linear)	4	691.23	705.24	-341.62	0 vs 1	46.90	<.001
2 (quadratic)	5	693.01	710.51	-341.50	1 vs 2	0.23	0.635
3 (cubic)	6	693.66	714.67	-340.83	2 vs 3	1.35	0.246
4 (quartic)	7	694.77	719.28	-340.38	3 vs 4	0.89	0.345
MMN amplitude - sigmoid model							
0 (Intercept only)	3	736.13	746.63	-365.07		-	-
Sigmoid model	5	664.65	682.15	-327.32	0 vs 1	75.48	<.001
P3 amplitude - linear and polynomial models							
0 (Intercept only)	3	716.43	726.93	-355.22		-	-
1 (linear)	4	717.46	731.46	-354.73	0 vs 1	0.97	0.324
2 (quadratic)	5	713.50	731.01	-351.75	1 vs 2	5.95	0.015
3 (cubic)	6	712.62	733.63	-350.31	2 vs 3	2.88	0.090
4 (quartic)	7	714.62	739.13	-350.31	3 vs 4	0.00	0.966

Note: df - degrees of freedom; AIC - Akaike information criterion; BIC - bayesian information criterion; logLik - log-likelihood; Test indicates which models (degrees) are compared in the test; L.Ratio - likelihood ratio; p - p-value of the likelihood ratio test.

Supplementary Table 5. Fitted models fixed effects.

	Value	SE	df
**MMN amplitude - polynomial model**			
Intercept	-0.83	0.1	206
Jitter (I)	6.51	0.9	206
Jitter (I) 2nd power	-0.43	0.9	206
Jitter (I) 3rd power	-1.04	0.9	206
Jitter (I) 4th power	-0.84	0.9	206
**MMN amplitude - sigmoid model**			
Asymptote (L)	-1.31	0.18	208
Inflection point (I_0)	-1.18	0.05	208
Slope at the inflection point (k)	6.24	1.73	208
**P3 amplitude - polynomial model**			
Intercept	0.76	0.10	206
Jitter (I)	-0.95	0.95	206
Jitter (I) 2nd power	-2.33	0.95	206
Jitter (I) 3rd power	1.60	0.95	206
Jitter (I) 4th power	0.04	0.95	206

Note: polynomial models utilized orthogonal polynomials; thus, for polynomial models of degree lower than 4, standard errors were the same as in the 4th degree model. Value - fixed effect estimate; SE - standard error; df - degrees of freedom.

Supplementary Table 6. R formulas used for fitting linear, polynomial and sigmoid models.

Model degree	Formula
MMN amplitude - linear and polynomial models	
0 (Intercept only)	mmn_amp ~ 1
1 (linear)	mmn_amp ~ poly(jitter, 1)
2 (quadratic)	mmn_amp ~ poly(jitter, 2)
3 (cubic)	mmn_amp ~ poly(jitter, 3)
4 (quartic)	mmn_amp ~ poly(jitter, 4)
MMN amplitude - sigmoid model	
0 (Intercept only)	mmn_amp ~ 1
Sigmoid model	mmn_amp ~ sigmoid(jitter, L, I_0, k)
P3 amplitude - linear and polynomial models	
0 (Intercept only)	p3_amp ~ 1
1 (linear)	p3_amp ~ poly(jitter, 1)
2 (quadratic)	p3_amp ~ poly(jitter, 2)
3 (cubic)	p3_amp ~ poly(jitter, 3)
4 (quartic)	p3_amp ~ poly(jitter, 4)

Note: For all models, participant id was inserted as random effect.

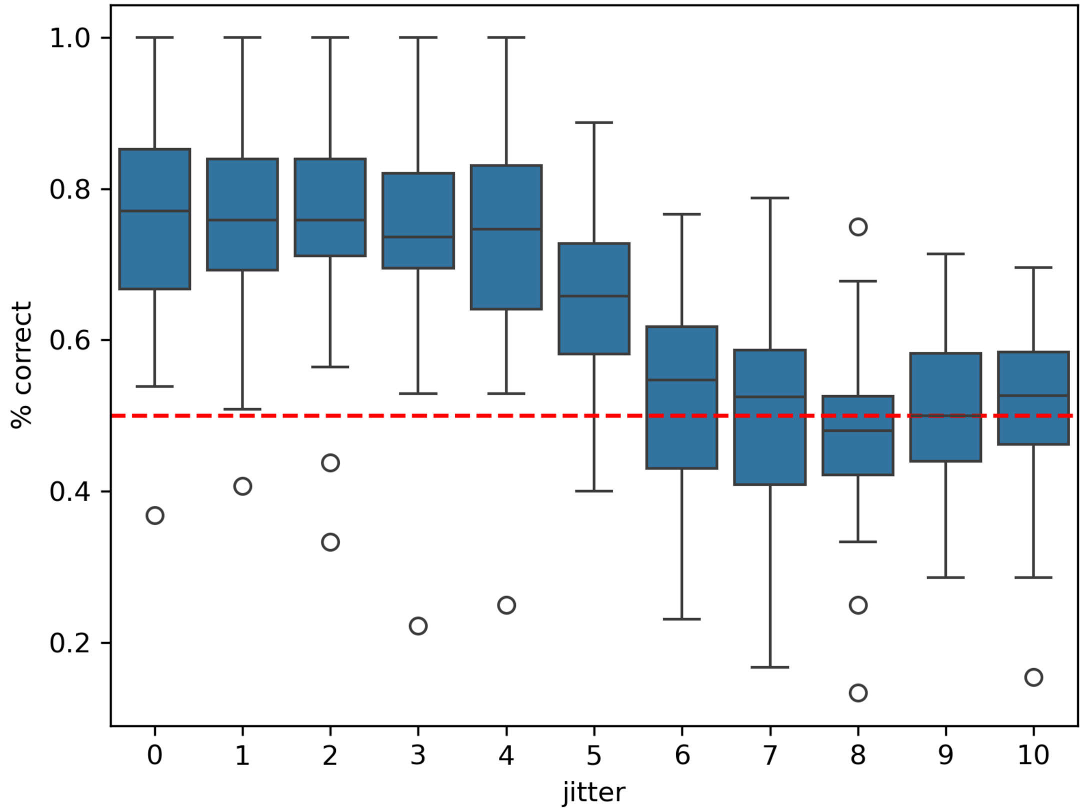

[Supplementary Figure 1. Results of the behavioral experiment. Figure shows percent-correct responses as a function of jitter. Boxes represent the range of quartiles 2 and 3, horizontal line within the box represents the median while whiskers indicate minima and maxima excluding outliers (shown as circles). Red dashed line indicates chance-level performance (50%).**]**

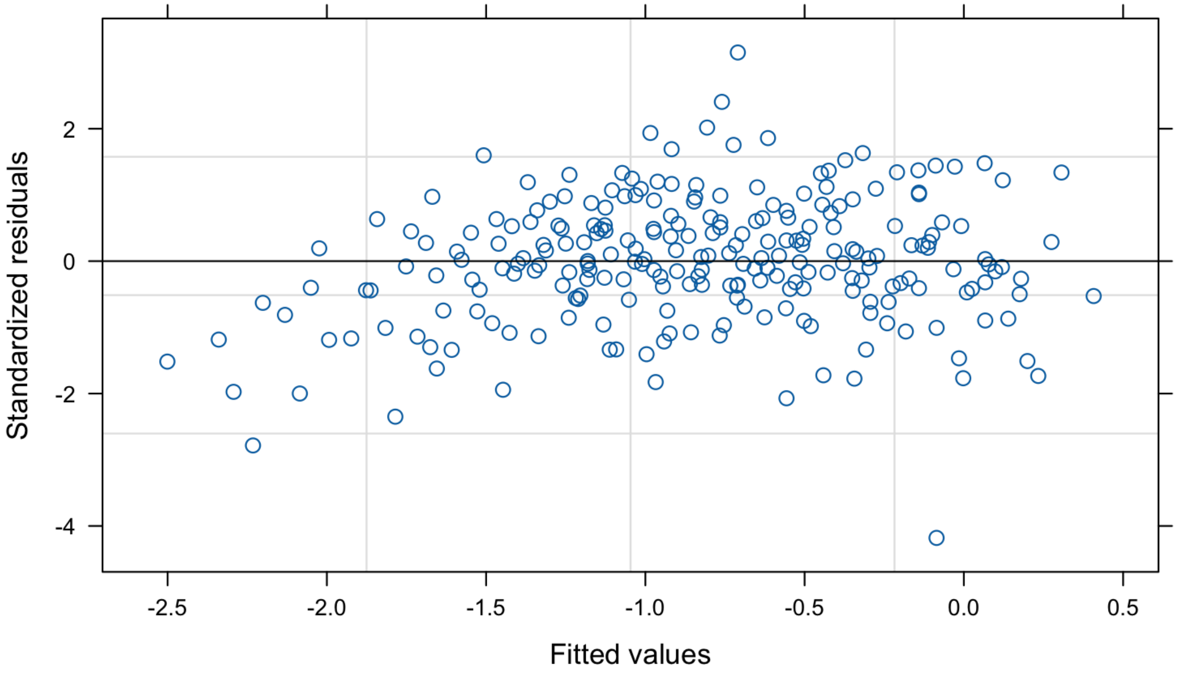

[Supplementary Figure 2. Residual plot for the linear model of MMN amplitude. Fitted values are presented on x-axis while standardized residuals are presented on y-axis.]

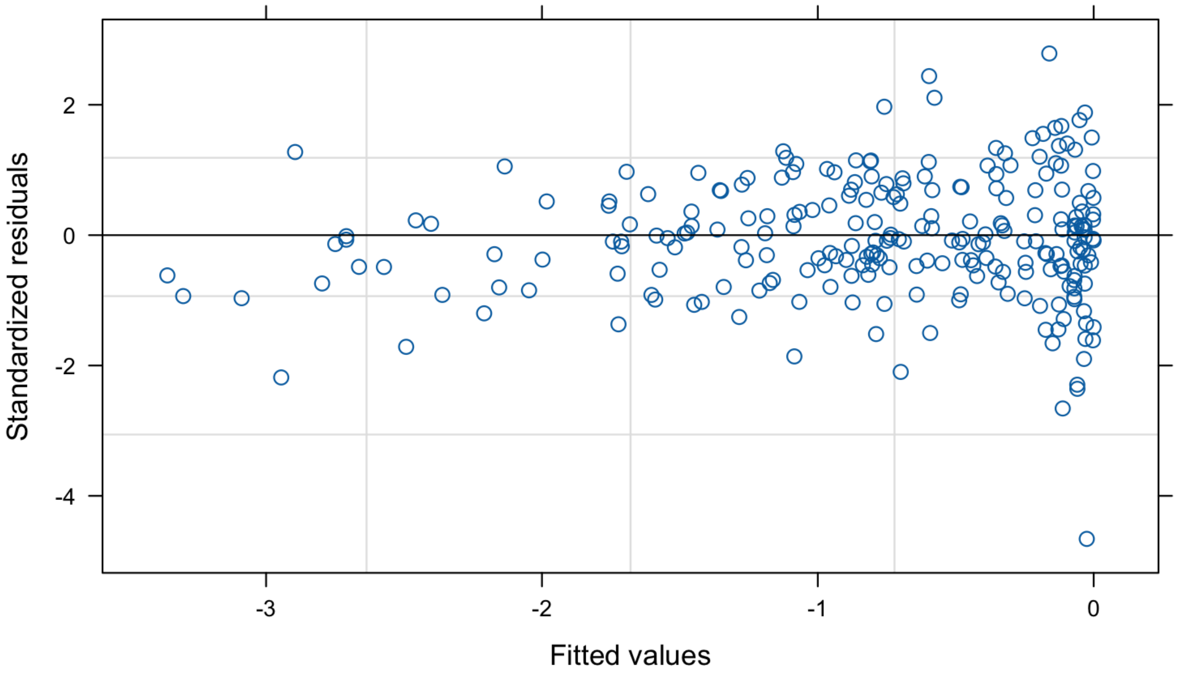

[Supplementary Figure 3. Residual plot for the sigmoid model of MMN amplitude. Fitted values are presented on x-axis while standardized residuals are presented on y-axis.]
